# The efficacy of extracorporeal photopheresis in the treatment of steroid refractory acute graft-versus-host disease: a systematic review and meta-analysis

**DOI:** 10.3389/fimmu.2025.1696862

**Published:** 2025-12-16

**Authors:** Zaid Muhanna, Ahmad Issa, Jehad Yasin, Leen Alkuttob, Maya Niss, Muaath Alsufi, Muntaser Al Zyoud, Shatha Farhan

**Affiliations:** 1 School of Medicine, The University of Jordan, Amman, Jordan; 2 Stem Cell Transplantation and Cellular Therapy, Henry Ford Health, Detroit, MI, United States

**Keywords:** acute graft-versus-host disease (aGVHD), steroid refractory acute graft-versus-host disease, extracorporeal photopheresis (ECP), systematic review, meta-analysis, meta-regression

## Abstract

**Introduction:**

Steroid-refractory acute graft-versus-host disease (SR-aGVHD) is a significant complication of hematopoietic stem cell transplantation (HSCT). Extracorporeal Photopheresis (ECP) represents a key second-line option. Previous reviews have provided valuable insights, and recent studies allow for an updated synthesis of efficacy, safety, and patterns of ECP use in SR-aGVHD, including outcomes not fully analysed previously. This study aims to address the literature gap by providing a comprehensive updated review of the efficacy of ECP and its patterns of use in SR-aGVHD.

**Materials and methods:**

A Systematic literature search was conducted as per PRISMA guidelines, up to September 2024, using the PubMed, Scopus, and Cochrane databases. Studies investigating the use of ECP in the setting of chronic GVHD, GVHD prophylaxis, or first-line treatment of aGVHD were excluded. Meta-analyses using fixed and random effects models were employed to estimate the pooled effect sizes.

**Results:**

Thirty-eight studies, including a total of 1249 participants, were included, and 29 were included in the quantitative analyses. Most studies focused on the adult population, and the majority used a retrospective single-arm study design (n = 30). Overall, skin, gut, and liver response rates were 72%, 89%, 54%, and 36%, respectively. The pooled steroid-sparing percentage was 66%. ECP showed significantly higher survival in patients with grade 2 GVHD compared with grades 3 and 4 (HR: 2.35, 95% CI: 1.67 – 3.29). ECP demonstrated a positive trend in overall survival compared to other treatments, but the results were not significant.

**Conclusion:**

This review indicates that ECP is an effective treatment for SR-aGVHD, with favorable response and survival outcomes. However, due to the heterogeneity observed in the analyses among the studies, more controlled trials are needed to establish its effects in combination with other agents and against other regimens.

**Systematic review registration:**

https://www.crd.york.ac.uk/prospero/, identifier CRD42024585471.

## Introduction

Hematopoietic stem cell transplantation (HSCT) is a vital procedure used to treat several hematological, immunological, and hereditary conditions, by revitalizing the immune system after high-dose chemotherapy or irradiation (1). Despite the advent of newer prophylactic regimens, including post-transplant cyclophosphamide (PTCy), 20-50% of HSCT patients develop acute graft-versus-host disease (aGVHD), a serious treatment complication of HSCT that significantly impacts patient survival (2–4). First-line treatment of aGVHD typically involves steroids, conventionally with prednisone or methylprednisolone at a starting dose of 1 to 2 mg/kg (5, 6). Unfortunately, a significant number of patients fail to respond to treatment, resulting in steroid-refractory aGVHD (SR-aGVHD), which is associated with a worse prognosis (7).

Extracorporeal photopheresis (ECP), an immunomodulatory cell therapy, is regarded as one of the main second-line treatment options for SR-aGVHD and involves exposing circulating leukocytes to 8-methoxypsoralen (8-MOP) and ultraviolet A (UVA) radiation upon reinfusion into the patient, which suppresses aGVHD through immunomodulatory pathways (8). These pathways promote a transition from pro- to anti-inflammatory cytokines, in addition to the upregulation of donor T regulatory cell (Treg) activity, suppressing alloreactive T-cells that mediate GVHD pathology (9, 10). Furthermore, ECP is associated with an excellent safety profile and limited immunosuppression, making it an ideal choice in this population and allowing its use alongside other agents (8). However, ECP is administered in multiple sessions across multiple weeks, posing a considerable burden to patients (9).

Regarding existing literature, two systematic reviews and meta-analyses (SR/MA) have examined the role of ECP in SR-aGVHD, by Zhang et al. and Abu-Dalle et al., both focusing mainly on prospective studies (11, 12), along with more recent narrative reviews by Greinix et al. and (8, 13, 17). These reviews have provided valuable insights, but the expanding body of clinical studies offers an opportunity for a comprehensive updated synthesis. Our objective is to evaluate the most recent evidence on the efficacy, safety, and patterns of use of ECP in SR-aGVHD, including outcomes that were not fully explored in earlier reviews.

## Materials and methods

### Protocol and registration

This systematic review was conducted in accordance with the Preferred Reporting Items for Systematic Reviews and Meta-Analyses (PRISMA) statement and registered in the international prospective register of systematic reviews (PROSPERO CRD42024585471).

### Search strategy

We conducted a systematic literature search across PubMed, Scopus, and the Cochrane Library for studies published from inception to September 2024, applying exclusion filters to remove non-human studies and non-English publications. Additionally, two reviewers screened the bibliographies of review articles and meta-analyses to identify any missed studies. The search strategy was adapted from a prior review on the same subject (14). A detailed description of the full-text search strategy is provided in **Supplementary Table 1**.

### Study selection

Studies were included if they focused on patients with steroid-refractory or steroid-dependent acute graft-versus-host disease (aGVHD) following allogeneic hematopoietic cell transplantation (HCT) from any source and evaluated extracorporeal photopheresis (ECP) as the intervention, with or without a comparator. Eligible studies must report at least one efficacy outcome such as overall response rate (ORR), complete, partial, or organ-specific response, duration of response (DOR), overall survival (OS), non-relapse mortality (NRM), and steroid-sparing effects. Accepted study designs included randomized controlled trials (RCTs), Interventional non-RCTs, case-control studies, and cohort studies. Studies were excluded if they were case reports, letters, comments, conference proceedings, or reviews. Studies were also excluded if they investigated ECP only as prophylaxis or first-line therapy.

Two independent reviewers initially screened the titles and abstracts of all retrieved articles for eligibility with any disagreement resolved through consensus. Subsequently, two other independent reviewers assessed the full-text articles of those selected during the initial screening. Any discrepancies between their findings were discussed and resolved until a consensus was reached.

### Data extraction and quality assessment

We developed a comprehensive data extraction form to ensure the systematic collection of all relevant clinical information. The extracted data included study characteristics such as design, sample size, number of treatment arms, treatment assignment, study phase, and treatment doses/regimens. Additionally, patient-related details were collected, including age, gender, pre-transplant diagnosis, transplant source, aGvHD grade, organ involvement, and conditioning regimen, along with key outcomes. To maintain accuracy, two independent reviewers conducted the data extraction, resolving any discrepancies through consultation with a third reviewer.

Quality assessment was carried out using appropriate tools based on the study design. For interventional studies the Methodological Index for Non-Randomized Studies (MINORS) was used (15). Observational studies with a control group were assessed using the Newcastle-Ottawa Scale (NOS), while a modified version was used for those without a control group (16). Two authors independently conducted the quality assessments, and any disagreements were resolved through discussion until a consensus was reached. We followed the guidelines for each assessment tool to evaluate the overall risk of bias in each study. Further details of the data extraction and quality assessment methods are discussed in the **Supplementary Material S1**; **Supplementary Material 9**–**11**).

### Statistical analysis

All statistical analyses were performed using R 4.3.3, utilizing the “meta”, “metafor”, and “metamedian” packages. Meta-analysis was conducted using both fixed-effect models (I² < 50%) and random-effects models (I² ≥ 50%). For proportional outcomes (e.g., response rates, steroid-sparing effects), the inverse variance method with the DerSimonian-Laird estimator was used to estimate the between-study variance (τ²). Confidence intervals for τ² and τ were calculated using the Jackson method. Additionally, the Freeman-Tukey double arcsine transformation was applied to stabilize variance and ensure that proportions were properly modeled. Subgroup analyses were conducted to explore differences in outcomes across categories (e.g., adult vs pediatric populations, treatment regimens).

For continuous outcomes (e.g., overall survival rates), hazard ratios (HRs) were pooled using the inverse variance method and the DerSimonian-Laird estimator for τ², with subgroup differences tested to explore potential moderators. The analysis of overall survival (OS) and non-relapse mortality (NRM) rates over time was stratified by year intervals (1, 2, and 4 years). The prediction interval for the random-effects model was derived based on the t-distribution, with degrees of freedom (df) set to 11 to account for variability among studies.

Meta-regression analysis was conducted using mixed-effects models for outcomes with sufficient study/arm numbers (k ≥ 10). This analysis aimed to investigate moderators, such as patient characteristics, treatment regimens, and publication year, on the primary outcomes/effect sizes. The results of the meta-regression are presented as univariate and multivariate models, with significance determined at p < 0.05.

Publication bias was assessed using Egger’s regression test and visualized through funnel plots. A significant bias was considered when the p-value of Egger’s test was below 0.05. To account for potential publication bias, the Trim and Fill method was applied, which adjusts the pooled estimates to correct for missing studies due to bias.

A meta-analysis of medians was also performed using the method of calculating the median of the difference of medians, weighted by sample size, to ensure the robustness of findings related to survival data. A 95% confidence level was applied for all median-based estimates. The analyses were considered significant if the p-value for the corresponding test was less than 0.05.

Heterogeneity across studies was assessed using the I² statistic, with values above 50% indicating substantial heterogeneity. Where appropriate, subgroup analyses were performed based on study characteristics, including patient age, GVHD grade, and ECP regimen.

All statistical tests were two-tailed, and a significance threshold of p < 0.05 was used for all analyses.

## Results

### Study selection

**Figure 1:**
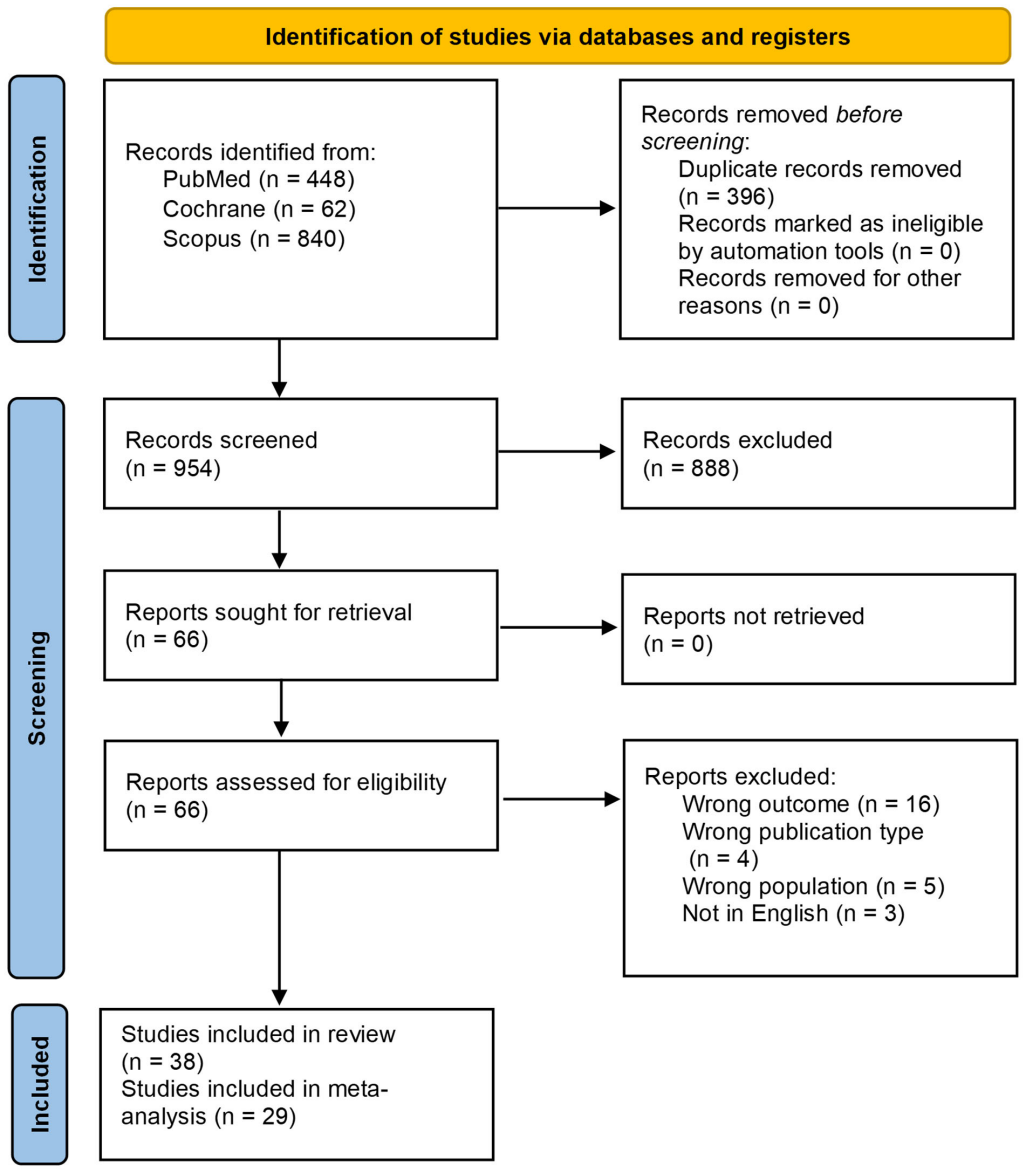
PRISMA diagram.

A total of 1350 records were identified through the initial database search. After excluding 396 duplicates, 888 records were further excluded through title & abstract screening. Full-text screening resulted in the exclusion of 28 records, including 16 studies due to having the wrong outcome, 4 for having the wrong publication type, 5 for having the wrong population, and 3 for being non-English studies. Ultimately, 38 studies were found to be eligible (17–54); however, 9 provided data that could not be used for the meta-analyses and so they were subsequently quantitatively excluded, resulting in the inclusion of 29 studies in the final meta-analyses. The PRIMSA flowchart is shown in **Figure 1**.

### Study characteristics

A total of 1249 patients were included in the study, with 30, 2, and 6 studies employing a retrospective cohort, prospective cohort, and a single-arm interventional study design, respectively (**Table 1**). Most studies were conducted on adult patients (n=23/38), with some studies involving both adults and pediatrics (n=5/38) (**Supplementary Table 2**). In-line ECP was the most commonly used technique (n=16/32), followed by off-line ECP (n=13/32) (**Supplementary Table 3**). Most studies reported on only steroid-refractory disease (n=19/32) with some also reporting on steroid-dependent (n=13/32) or steroid-intolerant or contraindicated disease (n=4/32) (**Supplementary Table 4**). The 1994 consensus conference criteria were the most used grading system (n=18/36) followed by the Glucksberg criteria (n=14/36) (55, 56), most studies were centered on grade II-IV GVHD with some also reporting on grade I GVHD (**Supplementary Table 4**). Conditions requiring transplant were reported in detail in the supplementary material (**Supplementary Table 5**), a more concise version is reported here with leukemia being the most common cause (**Table 1**). There was significant variation among the studies in terms of donor type, conditioning regimen, treatment combination, and treatment regimen, with the latter demonstrating considerable variation in terms of duration & frequency. (**Supplementary Table 3**, **6**–**8**).

**Table 1:** Baseline characteristics.

Author(s)	Type of study	Sample size	Median age (Years)	Lymphoma	Leukemia	MDS/MPN	Multiple myeloma & plasma cell disorders	Anemias & hemoglobinopathies	Other	Time to ECP (Weeks)	ECP duration (Weeks)
Besnier et al. (23) **	Retrospective Cohort	2	15	NR	NR	NR	NR	NR	NR	NR	NR
Greinix et al. (28)	Retrospective Cohort	6	NR	NR	NR	NR	NR	NR	NR	6.6 †	42
Smith et al. (50) **	Open-label, single-arm	6	29	NR	79	13	NR	8	NR	NR	5
Greinix et al. (29)	Open-label, single-arm	21	38	NR	86	NR	NR	NR	14	5.9 †	NR
Salvaneschi et al. (49)	Open-label, single-arm	9	10.3	NR	NR	NR	NR	NR	NR	5.1 ‡	NR
Messinaet al. (37)	Retrospective Cohort	33	9.6	3	81.8	NR	NR	NR	15.2	6.5 †	11.5
Garban et al. (26)	Prospective Cohort	12	40	NR	75	17	8	NR	NR	NR	NR
Greinix et al. (30)	Open-label, single-arm	38	40	NR	NR	NR	NR	NR	NR	4.9 †	NR
Berger et al. (21)	Retrospective Cohort	15	10.8	7	67	7	NR	13	7	5.4 †	2.8
Kanold et al. (33)	Open-label, single-arm	12	13.5	8.3	58.4	NR	NR	33.3	NR	6.5 ‡	NR
Calore et al. (24)	Prospective Cohort	23	9.6	NR	56.5	21.7	8.7	8.7	4.3	6.1 †	24.4
Perfetti et al. (44)	Retrospective Cohort	15	41	7	94	NR	NR	NR	NR	8 ‡	7
González Vicent et al. (27) *	Retrospective Cohort	21	10	4	70	NR	NR	14	11	4.3	27.1
Perotti et al.(2010) ***(45)	Retrospective Cohort	50	9.9	NR	66	NR	NR	NR	34	1.3 ‡	NR
Hautmann et al. (31)	Retrospective Cohort	30	42	NR	84	3	NR	NR	13	6.4 †	7.1
Jagasia et al. (32)	Retrospective Cohort	57	57	NR	NR	NR	NR	NR	NR	6.4 †	6.4
Rubegni et al. (47)	Retrospective Cohort	9	49.6	22	55	NR	22	NR	NR	6.6 ‡	NR
Ussowicz et al. (52)	Retrospective Cohort	8	20.5	12.5	75	NR	NR	12.5	NR	3.3 †	14
Das-Gupta et al. (25)	Retrospective Cohort	128	41.9	NR	NR	NR	NR	NR	NR	6 †	8.6
Berger et al. (22)	Retrospective Cohort	34	12	9	60	6	NR	NR	24	5.4 †	6.7
Malagola et al. (36)	Retrospective Cohort	45	SR: 47, SD: 45	17.8	51.1	13.3	13.3	NR	6	NR	No Response: 11, PR: 27.5
Niittyvuopio et al. (40)	Retrospective Cohort	52	50	13.5	52.1	23.1	11.5	NR	NR	1.5	NR
Nygaard et al. (41)	Retrospective Cohort	38	56	NR	NR	NR	NR	NR	NR	5.3 ‡	NR
Sakellari et al. (48)	Retrospective Cohort	19	44	21	52	10	16	NR	NR	1.6 ‡	NR
Worel et al. (54)	Retrospective Cohort	99	41	NR	80	7	NR	NR	13	5 †	4
Winther-Jørgensen et al. (53)	Retrospective Cohort	9	7	11	55	NR	NR	22	11	9.4 †	17
Axt et al. (19) ***	Retrospective Cohort	13	NR	NR	NR	NR	NR	NR	NR	NR	NR
Oarbeascoa et al. (42)	Retrospective Cohort	65	49	NR	49	9	NR	NR	42	2.9 ‡	NR
Modemann et al (39)	Retrospective Cohort	18	58.5	NR	34	61	6	NR	NR	12.4 †	22.8
Batgi et al.(2021) ***(20)	Retrospective Cohort	75	36	5.2	73.3	4	2.6	2.6	12	11.14 ‡	NR
Kitko t al. (35)	Open-label, single-arm	29	8	NR	58.6	NR	NR	NR	41.4	1	NR
Reschke et al. (2022) ** (46)	Retrospective Cohort	9	59	33.3	11.11	33.3	22.2	NR	NR	3 ‡	11.43
Canto et al. (17)	Retrospective Cohort	29	8	NR	89.6	NR	NR	3.4	6.8	NR	NR
Kaya et al. (34)	Retrospective Cohort	35	36.1	6	65	NR	3	27	NR	Survived: 7, Dead: 5 ‡	Survived: 12, Dead: 6
Penack et al. (43)	Retrospective Cohort	53	52.6	9.4	64.1	26.4	NR	NR	NR	4.1 ‡	9.1
Solh et al. (51)	Retrospective Cohort	79	NR	19	48	29	NR	NR	4	8.14 ‡	NR
Canto et al. (18)	Retrospective Cohort	28	48.7	17.8	71.4	10.7	3.6	NR	NR	NR	12
Michallet et al.(2024) *(38)	Retrospective Cohort	25	52	3.5	58	26.5	12	NR	NR	6.5 ‡	NR

**ECP,** Extracorporeal photopheresis; **MDS,** Myelodysplastic Syndromes; **MPN,** Myeloproliferative neoplasms; **SD,** Steroid Dependent; **SR,** Steroid refractory; **PR,** Partial Response; **NR,** Not reported; **G,** Grade.

(*) Studies included both chronic and acute GVHD.

(**) Studies evaluated response in only one organ.

(***) Studies included overlap syndromes.

(†) Post stem cell transplant

(‡) Post GVHD diagnosis

A detailed overview of the study characteristics and the quality assessment of the individual studies are provided in the supplementary material. (**Supplementary Table 2**, **9**–**11**).

### Meta analyses

#### Response rate

**Figure 2:**
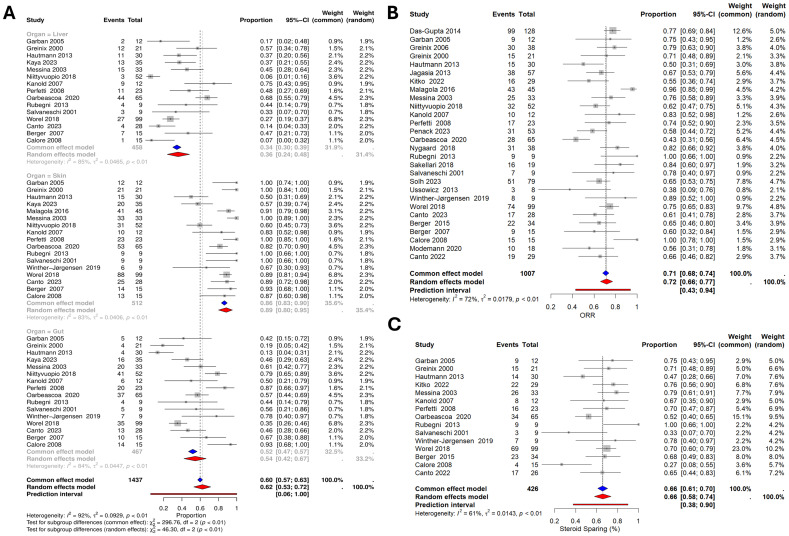
Forest plots of response rates **(A)** Organ-specific response rate. **(B)** Overall response rate (ORR). **(C)** Steroid-sparing rate.

The ORR was pooled using a total of 28 studies (1007 patients), resulting in a pooled ORR of 72% (95% CI: 68% - 77%; **Figure 2B**). For the organ-specific responses, 17 studies (512 patients), 16 (467 patients), and 15 (458 patients) were included for the skin, gastrointestinal tract, and liver responses, respectively. The response rate for the skin was 89% (95% CI: 80% - 95%), the gastrointestinal tract was 54% (95% CI: 42%- 67%), and the liver was 36% (95% CI: 24% - 48%) (**Figure 2A**). Additionally, the pooled steroid-sparing rate was 66% (95% CI: 58% - 74%; 15 studies, 426 patients) (**Figure 2C**). Substantial heterogeneity was noted across the analyses, with I^2^ of 84%, 59%, and 54% for liver, skin, and gut responses, respectively. Similarly, ORR and the steroid-sparing rate showed significant heterogeneity (I² = 72% and 61%, respectively). (**Figures 2A-C**).

#### Survival

**Figure 3:**
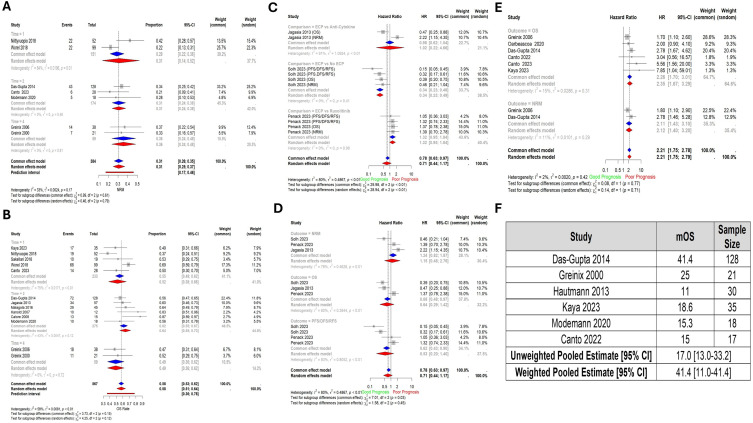
Forest plots of survival outcomes **(A)** Non-relapse mortality by year (NRM). Time – Year **(B)** Overall survival rate by year. Time – Year **(C)** Pooled comparisons of ECP against other drugs by type of drug. Each of PFS, DFS, and RFS is represented under the same surrogate “PFS/DFS/RFS”. **(D)** Pooled comparisons of ECP against other drugs by outcome. **(E)** Pooled Hazard Ratio of Grade 3 and 4 Graft-versus-Host Disease compared to Grade 2. **(F)** Pooled median overall survival. Abbreviations: NRM – Non-relapse mortality; OS – Overall Survival; PFS – Progression-free survival; FFS – Failure-free survival; DFS – Disease-free survival; Time – Year; No ECP – Same regimen with the exclusion of ECP.

The NRM at 1, 2, and 4 years was 31% (95% CI: 14% - 52%; 2 studies, 151 patients), 31% (95% CI: 24% - 38%; 3 studies, 174 patients), and 36% (95% CI: 24% - 48%; 2 studies; 59 patients), respectively (**Figure 3A**). Furthermore, the pooled OS rate at year 1 was 52% (95% CI: 38% - 66%), at year 2 64% (95% CI: 55% - 73%), and at year 4 was 49% (95% CI: 36% - 62%), and they included 5 studies (233 patients), 6 studies (275 patients), and 2 studies (59 patients) respectively (**Figure 3B**).

When ECP was compared to other treatments, there was no significant difference in terms of pooled prognostic outcomes (HR: 0.71, 95% CI: 0.44 - 1.17; 3 studies, 189 patients), or at the level of individual outcomes (NRM, OS, and PFS/DFS/RFS). However, when compared against specific comparators, ECP versus No ECP (HR: 0.34, 95% CI: 0.23-0.49); was significant (HRs for OS, NRM, and PFS/RFS/DFS pooled) (**Figure 3C-D**).

The pooled HR comparing OS between patients with grade II vs grade III and IV aGVHD showed a significantly worse OS in the latter group (HR: 2.35, 95% CI: 1.67 – 3.29; 6 studies, 323 patients) (**Figure 3E**).

The pooled median OS in the weighted model was 41.1 months (95% CI: 11.0-41.1) (**Figure 3F**).

#### Meta-regression analysis

Multiple meta-regression outcomes are summarized in **Table 2**. The table highlights significant heterogeneity across studies (I² values), with some outcomes showing strong moderator effects. Key findings include the influence of population type (Pediatrics vs. Mixed population vs. Adults), with studies focusing on pediatric populations showing superior survival, and studies containing mixed populations showing improved skin and gut responses. Additionally, a positive trend was seen in skin response with publication year, with more recent studies showing improved response. Some outcomes, like liver response and steroid-sparing, lack significant moderators, implying unexplained variability.

**Table 2:** Multiple Meta-Regression results. Combination: combination with other active treatments (yes/no). Reference categories: combination (No), Comparison (ECP vs Anti-Cytokine), Population (Adults). Variables included in models: publication year, classification (Glucksberg, MAGIC, etc.), combination, outcome (OS, NRM, PFS/RFS/DFS), and comparison.

Outcome	k (Studies)	I² (%)	R² (%)	Test for residual heterogeneity (QE, p-value)	Test of moderators (QM, p-value)	Significant outcomes (p < 0.05)
Liver Response	15	83.69	0.00	42.9159, p < 0.0001	3.8258, p = 0.7996	None
Skin Response	17	58.77	68.20	21.8310, p = 0.0094	28.4061, p = 0.0002	Population: Pediatrics/Adults (Mixed) (-0.4271, p = 0.0117) Publication Year (-0.0273, p < 0.0001)
Gut Response	16	53.91	73.74	17.3558, p = 0.0266	28.0059, p = 0.0002	Population: Pediatrics/Adults (Mixed) (-0.6178, p = 0.0002) Combination: Yes (0.3290, p = 0.0008)
NRM	17	60.85	17.05	22.9901, p = 0.0062	12.0717, p = 0.0982	None
HR (ECP vs Treatment)	10	41.09	80.15	8.4871, p = 0.1314	20.4411, p = 0.0004	Comparison: ECP vs No ECP (-0.9441, p = 0.0307)
Steroid Sparing	15	68.43	0.00	19.0079, p = 0.0042	5.0247, p = 0.7549	None
ORR	28	73.01	0.00	70.3870, p < 0.0001	6.8987, p = 0.5476	None
OS Rate	14	72.33	0.00	46.9791, p < 0.0001	7.0503, p = 0.4237	Population: Pediatrics (0.2586, p = 0.0483)

#### Publication bias

**Figure 4:**
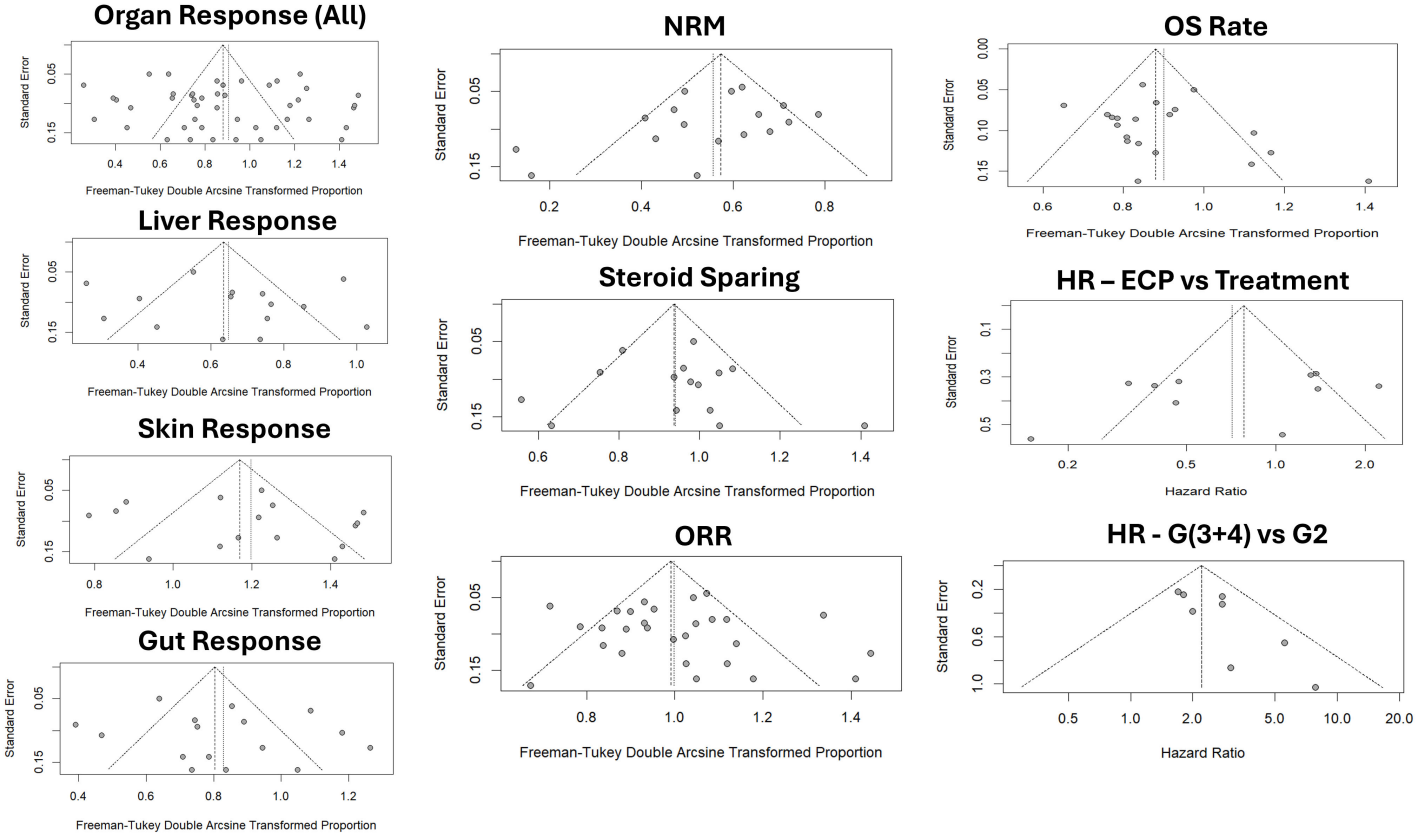
Funnel plots Abbreviations: NRM – Non-relapse mortality; OS – Overall Survival; ORR – Overall response rate; HR – Hazard ratio; G2 – Grade 2 GVHD; G(3 + 4) – Grade 3 and 4 GVHD.

No significant publication bias was observed except for HR comparing patients with grade II vs grade III and IV aGVHD (bias estimate = 1.5917, p = 0.0414) (**Table 3**). Funnel plots are displayed in **Figure 4**.

**Table 3:** Egger’s regression test results for all outcomes.

Category	Test result (t)	df	p-value	Bias estimate	SE
Organ Response - All	0.8	46	0.426	1.173	1.4605
Skin	0.84	15	0.416	1.4343	1.7146
Liver	0.34	13	0.7375	0.6817	1.9904
Gut	0.73	14	0.4774	1.2993	1.7796
NRM	-1.29	15	0.2179	-1.4467	1.1248
Steroid Sparing	0.17	13	0.8643	0.2145	1.2303
ORR	0.53	26	0.6005	0.5453	1.0287
OS Rate	1.66	19	0.1129	1.6669	1.0029
HR – Grade	2.59	6	**0.0414**	1.5917	0.6153
HR – ECP vs Treatment	-1.14	8	0.2856	-3.9872	3.4847

Numbers in bold indicate a significant value.

## Discussion

This study synthesized data from 38 studies comprising 1249 patients, of which 29 studies (1007 patients) were included in the meta-analyses. Unlike prior meta-analyses which were limited to prospective trials (11, 12), this study incorporated a wider array of data, including retrospective studies. This broader inclusion strategy improves the generalizability of the findings and allows for the analysis of novel outcomes such as time to ECP, treatment duration, and steroid-sparing effects, factors with important implications for clinical decision-making.

The pooled ORR to ECP was 72%, with a steroid-sparing effect observed in 66% of patients. These findings reinforce the role of ECP as a valuable second-line therapy for aGVHD, especially with the high steroid-sparing effect. Organ-specific responses varied significantly: skin involvement demonstrated the highest response rate (89%), while gastrointestinal (54%) and hepatic (36%) responses were more modest. These differences show the variable sensitivity of target organs to ECP and may have implications for tailored treatment strategies, which is consistent with the literature (8). This is likely due to the distribution and trafficking patterns of alloreactive T cells in skin versus gut/liver. In cutaneous GVHD, many of the pathogenic T cells bear skin-homing markers and recirculate through the peripheral blood en route to the skin. This means a larger fraction of skin-targeting effector T cells is accessible during ECP leukapheresis. By contrast, GVHD effectors that home to the gut or liver may reside more sequestered in those tissues, making them less exposed to ECP’s direct pro-apoptotic effects (57).

NRM remained relatively stable at around 31–36% at years 1, 2 and 4. This suggests that mortality rates due to causes other than relapse are similar and does not drastically increase over time as evident by the overlapping CI. This trend aligns with earlier findings (25). Furthermore, pooled OS increased from year 1 to year 2 (52% → 64%), which is consistent with previously reported data (51) and then declined slightly at year 4 (49%), possibly indicating late mortality due to relapse or complications. The decline after year 2 may reflect late post-treatment effects, disease progression, or comorbidities. Patients with grade III/IV aGVHD had significantly worse survival than those with grade II (HR: 2.35; 95% CI: 1.67–3.29). This indicates that advanced-grade disease nearly doubles the risk of death.

There were few comparative studies; however, the pooled results showed that ECP was not significantly different from pooled comparators overall (HR: 0.71; 95% CI: 0.44–1.17) (43, 51). However, when ECP was directly compared to “No ECP”, it was significantly beneficial (HR: 0.34; 95% CI: 0.23–0.49). This indicates that ECP outperforms not receiving ECP, but its effect may be diluted when compared against other active therapies. In addition, a significant number of studies combined ECP with other active treatments, while others did not, and the meta-regression results showed that it did not affect the outcomes, with the exception of improved gut response in patients receiving active combinations (**Table 2**). However, these results were most likely influenced by the variations in the type of combinations and the number of patients who received such combinations from the entire population.

The limitations of this study include the retrospective nature of most of the included studies. Retrospective studies inherently have a higher risk of bias since the data collection, data entry, and data quality assurance were not planned ahead of time (58). Additionally, some clinical outcomes (such as ORR, complete, partial, or organ-specific response, DOR, OS, NRM, and steroid-sparing effects) were inconsistently reported or entirely absent, which might prevent a full assessment of the clinical impact of ECP. Furthermore, there was considerable clinical variation across studies regarding grading systems used to define aGVHD, ECP regimens, baseline characteristics, treatment duration and adjunctive therapies which may influence the variability of pooled estimates.

The meta-analysis exhibits considerable heterogeneity, particularly for liver response, ORR, and OS rate, as reflected by high I² values and significant residual heterogeneity. Some of these variations are explained by moderators such as population type (adults vs. mixed) and publication year for skin and gut responses. Interestingly, the pediatric population was associated with improved OS outcomes, indicating potential age-related variability in response. This finding contrasts with earlier reports that did not identify age as a significant predictor (59). However, for outcomes like liver response and steroid-sparing, none of the tested moderators could explain the heterogeneity, indicating unexplained differences, the heterogeneity in the latter could be explained by the lack of consistency in how it was defined across different studies. Additionally, publication bias was observed in HR comparing patients with grade II vs those with grade III and IV aGVHD and it may contribute to heterogeneity in this outcome.

While ECP offers a valuable second-line treatment for SR-aGVHD, its optimal role remains undefined due to a lack of standardized procedures and reporting. Future studies should prioritize investigating ECP in treatment regimens alongside other agents such as Ruxolitinib and comparative studies to further explore its optimal use. A global, prospective registry or randomized comparative studies may be essential to resolve current uncertainties and guide the use of ECP in SR-aGVHD.

## Data Availability

The original contributions presented in the study are included in the article/**Supplementary Material**. Further inquiries can be directed to the corresponding author.
