# Supplementary material for: The efficacy of extracorporeal photopheresis in the treatment of steroid refractory acute graft-versus-host disease: a systematic review and meta-analysis
Source: Front Immunol. 2025 Dec 16;16:1696862. doi: 10.3389/fimmu.2025.1696862 (PMC12747844; doi:10.3389/fimmu.2025.1696862)
Supplement: Supplementary file 1 [file DataSheet1.docx]

Contents

[**Supplementary Table 1 – Search Strategy (Table S1)** 2](#_Toc202294473)

[**Methodology (Item S1)** 3](#_Toc202294474)

[**Supplementary Table 2 – Baseline Characteristics (Table S2)** 4](#_Toc202294475)

[**Supplementary Table 3 – Technique & Schedule (Table S3)** 6](#_Toc202294476)

[**Supplementary Table 4 – Response to Steroids & Grade (Table S4)** 7](#_Toc202294477)

[**Supplementary Table 5 – Primary Disease (Table S5)** 8](#_Toc202294478)

[**Supplementary Table 6 – Conditioning Regimen (Table S6)** 9](#_Toc202294479)

[**Supplementary Table 7 – Treatment Combination (Table S7)** 10](#_Toc202294480)

[**Supplementary Table 8 – Donor Type & Source (Table S8)** 11](#_Toc202294481)

[**Supplementary Table 9 – Quality assessment for controlled retrospective cohort studies (Table S9)** 12](#_Toc202294482)

[**Supplementary Table 10 – Quality assessment for single-arm retrospective cohort studies (Table S10)** 13](#_Toc202294483)

[**Supplementary Table 11 – Quality assessment for single-arm interventional studies (Table S11)** 14](#_Toc202294484)

| "Photopheresis"[MeSH] OR photopheresis OR extracorporeal photopheresis OR ECP[tiab] OR Extracorporeal Photochemotherapy OR Extracorporeal Photochemotherapies OR Photochemotherapies, Extracorporeal | 1 |
| --- | --- |
| graft versus host disease OR graft vs host disease OR acute graft versus host disease OR acute graft vs host disease OR GvHD OR aGvHD OR graft versus host reaction OR graft vs host reaction OR "Graft vs Host Disease"[Mesh] | 2 |
| #1 AND #2 | 3 |
| Systematic Review[Publication Type] | 4 |
| Review[Publication Type] | 5 |
| #3 NOT #4 NOT #5 | 6 |

# **Supplementary Table 1 – Search Strategy (Table S1)**

Search strategy for Medline is presented; search strategies for other databases were modified according to each database.

# **Methodology (Item S1)**

**Data extraction and quality assessment**

The response to ECP was reported at 2 months; if unavailable, then the 3-month time point was used. If neither were found, then the best response to treatment was reported. In addition, grade was reported as both the maximum grade and the grade at the start of treatment; if the distinction was unclear, it was considered to be the maximum grade. Furthermore, in studies where outcomes for the upper and lower gastrointestinal tract (GI) were reported, only the lower GI was considered. In studies where patients were classified into partial response to steroids and no response to steroids, the former was reported as steroid dependent, while the latter was reported as steroid refractory.

Treatment combinations in this study and during the analyses were defined into three categories: the first included patients treated with ECP and other agents that were not strongly active, the second included patients treated with ECP and active agents, and the third included patients whose combination of drugs was not clear. The distinction between the first two groups was mostly arbitrary.

For the purposes of the quality assessment, an adequate follow-up was set at 6 months, and the comparability of the cohorts was based on age, gender, the involvement of organs, and staging.

| Author(s) | Year of Publication | Type of Study | Population | Duration of Study | Location of Study | Median Follow-Up (Months) |
| --- | --- | --- | --- | --- | --- | --- |
| Besnier | 1997 | Retrospective Cohort | Pediatrics, Adults | 13 | Italy | NR |
| Greinix | 1998 | Retrospective Single | Adults | 2 | Austria | 5 |
| Smith | 1998 | Single-arm interventional | Adults | 6 | USA | NR |
| Greinix | 2000 | Single-arm interventional | Adults | 5 | Austria | NR |
| Salvaneschi | 2001 | Single-arm interventional | Pediatrics | 3 | Italy | 8 |
| Messina | 2003 | Retrospective Single | Pediatrics | 9 | Italy | NR |
| Garban | 2005 | Prospective Single | Adults | 7 | France | NR |
| Greinix | 2006 | Single-arm interventional | Adults | 9 | Austria | 52 |
| Berger | 2007 | Retrospective single | Pediatrics | 5 | Italy | 19.2 |
| Kanold | 2007 | Single-arm interventional | Pediatrics | 10 | France | 16 |
| Perfetti | 2008 | Retrospective single | Adults | 11 | Italy | 37 |
| Calore | 2008 | Prospective Cohort | Pediatrics | 7 | Italy | NR |
| González Vicent | 2010 | Retrospective Single | Pediatrics | 5 | Spain | 5.6 |
| Perotti | 2010 | Retrospective single | Pediatrics | 10 | Italy | 14 |
| Hautmann | 2013 | Retrospective Single | Pediatrics, Adults | 12 | Germany | 23 |
| Jagasia | 2013 | Retrospective Cohort | Pediatrics, Adults | 7 | USA/UK/Austria/France | 37.9 |
| Rubegni | 2013 | Retrospective single | Adults | 7 | Italy | 3 |
| Ussowicz | 2013 | Retrospective single | Adults | 7 | Poland | 20.8 |
| Das-Gupta | 2014 | Retrospective Single | Adults | 16 | USA/UK/Austria | 23.3 |
| Berger | 2015 | Retrospective single | Pediatrics, Adults | 13 | Italy | S = 48, D = 7 |
| Malagola | 2016 | Retrospective Single | Adults | 10 | Italy | 20 |
| Niittyvuopio | 2018 | Retrospective Single | Adults | 13 | Finland | 6 |
| [Nygaard](https://pubmed.ncbi.nlm.nih.gov/?term=Nygaard+M&cauthor_id=29907805) | 2018 | Retrospective single | Adults | 4 | Denmark | 10.4 |
| Sakellari | 2018 | Retrospective single | Adults | 15 | Greece | 17.5 |
| Worel | 2018 | retrospective | Adults | 14 | Austria | NR |
| Winther-Jørgensen | 2019 | Retrospective single | Pediatrics | 5 | Denmark | NR |
| Axt | 2019 | Retrospective single | Adults | 10 | Germany | NR |
| Oarbeascoa | 2020 | Retrospective single | Adults | NR | Spain | 24 |
| Modemann | 2020 | Retrospective Cohort | Adults | 3 | Germany | 29.6 |
| Batgi | 2021 | Retrospective single | Adults | NR | Turkey | 6 |
| Kitko | 2022 | Single-arm interventional | Pediatrics | 3 | USA | NR |
| Reschke | 2022 | Retrospective single | Adults | 10 | Germany | NR |
| Canto | 2022 | Retrospective single | Pediatrics | 9 | Spain | 13.3 |
| Kaya | 2023 | Retrospective Single | Adults | 14 | Turkey | NR |
| [Penack](https://pubmed.ncbi.nlm.nih.gov/?term=Penack%20O%5BAuthor%5D) | 2023 | Retrospective cohort | Adults | 3 | Europe | 43.7 |
| Solh | 2023 | Retrospective Cohort | Pediatrics, Adults | 9 | USA | 42.2 |
| Canto | 2023 | Retrospective single | Adults | 8 | Spain | 36 |
| Michallet | 2024 | Retrospective Single | Adults | 15 | France | 21 |

# **Supplementary Table 2 – Baseline Characteristics (Table S2)**

**NR -**  Not Reported; **S -**  Survived; **D -** Deceased

| Author(s) | ECP Technique | Regimen |
| --- | --- | --- |
| Besnier | In-line | ECP was performed 3 times a week for 3 weeks. |
| Greinix | In-line | ECP performed twice biweekly for 3 months, then reduced to twice every 4 weeks until resolution. |
| Smith | In-line | ECP performed 2 to 3 times weekly until response was achieved, then tapered. |
| Greinix | In-line | ECP given twice every 1 to 2 weeks. If there is a response, frequency is reduced to twice every 2 to 4 weeks until maximal response, followed by tapering. |
| Salvaneschi | Off-line | ECP given three times per week on alternate days until improvement, then responding patients received ECP twice biweekly on consecutive days for 3 months, followed by tapering. |
| Messina | In-line | ECP performed on two consecutive days weekly for the first month, then every two weeks for another 2 months, and then monthly for at least 3 additional months. |
| Garban | Off-Line | Six ECP sessions given over 3 weeks. If the patient achieves complete response treatment is stopped. If partial response is achieved, ECP continues once weekly until complete response. If no response, treatment is stopped |
| Greinix | In-line | ECP administered twice weekly until maximal response is reached, then stopped without tapering. |
| Berger | In-line, Off-line | ECP was performed on two consecutive days at weekly intervals for 1 month, followed by biweekly sessions for the second and third months, then monthly for an additional 3 months. |
| Kanold | Off-line | ECP performed three times per week for 3 weeks, with a one day interval in-between then tapering for patients who showed improvement or were stabilized. |
| Perfetti | Off-line | ECP given twice weekly on consecutive days for 1 month, then every second week for 2 months, followed by twice monthly until complete response or stabilization. |
| Calore | Off-line | ECP was performed on two consecutive days at weekly intervals for 1 month, followed by biweekly sessions for the second and third months, then monthly for an additional 3 months. |
| González Vicent | Off-Line | ECP performed twice weekly until clinical improvement. |
| Perotti | Off-line | ECP performed 2 to 3 times per week on alternate days until improvement, then reduced to twice weekly for 2 weeks, followed by twice biweekly for 3 cycles, and then twice per month based on clinical improvement or tapering of immunosuppression. |
| Hautmann | Off-Line | ECP administered 2 to 3 times per week until partial or complete response, then reduced to twice weekly every two week, followed by twice per month if improvement continues. |
| Jagasia | N/A | ECP given 2 to 3 times weekly or biweekly until maximal response, then discontinued or tapered. |
| Rubegni | In-line | ECP performed twice weekly for consecutive days at one week intervals until improvement, then biweekly, followed by tapering. |
| Ussowicz | In-line | ECP given on two consecutive days biweekly for 14 weeks. |
| Das-Gupta | N/A | ECP administered 2 to 3 times per week for 4 to 6 weeks, then reduced to every other week. After maximal response, treatment stopped or tapered. |
| Berger | In-line, Off-line | ECP given twice weekly on consecutive days for 1 month, then twice biweekly for 2 or 3 months, followed by twice monthly for 3 months. |
| Malagola | Off-line | Two cycles of procedures were performed weekly for the first month, then, every other week for the following 4 weeks and then monthly, until permanent steroid discontinuation with complete response. |
| Niittyvuopio | In-line | ECP administered twice weekly, avoiding consecutive days, except for one patient who received consecutive-day treatment, until maximum response was achieved. |
| [Nygaard](https://pubmed.ncbi.nlm.nih.gov/?term=Nygaard+M&cauthor_id=29907805) | In-line | ECP given weekly in 35 cases, and biweekly in 3 cases. |
| Sakellari | In-line | ECP was administered twice weekly for 1 month, then once biweekly for 3 months, followed by once monthly for 6 months, with tapering. |
| Worel | In-line | ECP performed 2 to 3 times weekly or biweekly, with discontinuation if complete response was achieved or if steroid dose fell below 0.5 mg/kg. |
| Winther-Jørgensen | In-line | ECP administered on consecutive days weekly, then reduced to once biweekly for 2 to 3 months, followed by discontinuation or tapering to once per month for up to 3 months. |
| Axt | N/A | N/A |
| Oarbeascoa | Off-line | ECP was administered twice weekly for 4 weeks, then once every 2 weeks for at least 4 weeks. If response was achieved, treatment was either discontinued after steroid withdrawal or continued based on institutional protocols. In 25% of cases, ECP was administered once per week for 4 weeks, then every 2 weeks for 4 weeks, then monthly, with response-based discontinuation. |
| Modemann | In-line | Two ECP sessions were administered weekly for 2 weeks, with tapering. For most patients, subsequent ECP was conducted weekly. |
| Batgi | Off-line | ECP administered on two consecutive days biweekly until signs resolved. If complete response was achieved, frequency was reduced from twice to once biweekly, with further adjustments based on response. |
| Kitko | N/A | ECP was administered three times per week for 4 weeks, then twice weekly for 5 to 12 weeks. |
| Reschke | In-line | ECP administered twice weekly on non-consecutive treatment days until maximum response. |
| Canto | In-line, Off-line | ECP given twice weekly for acute GVHD until response, then once weekly until maximal response, followed by biweekly sessions. |
| Kaya | Off-line | Four ECP sessions performed with a minimum interval of one week, on two consecutive days with two days of no action between treatments. |
| [Penack](https://pubmed.ncbi.nlm.nih.gov/?term=Penack%20O%5BAuthor%5D) | N/A | N/A |
| Solh | N/A | ECP initiated with 25 treatments: three times in the first week, then twice weekly. If response was achieved but not complete, maintenance treatment was given every other week for 24 additional treatments. |
| Canto | Off-line | ECP performed twice per week for 2 weeks, then once per week for 2 weeks, followed by biweekly sessions for 5 months. Tapering was based on response and immunosuppressant adjustments. |
| Michallet | In-line | ECP given twice per week for 2 weeks, then once per week for 4 weeks. |

# **Supplementary Table 3 – Technique & Schedule (Table S3)**

**N/A** – Not Available

| Author(s) | SR % | SD % | SI% | Classification System | G1 | G2 | G3 | G4 | G1 | G2 | G3 | G4 | A | B | C | D |
| --- | --- | --- | --- | --- | --- | --- | --- | --- | --- | --- | --- | --- | --- | --- | --- | --- |
| Besnier | 100 | 0 | 0 | Consensus Conf. | N/A | N/A | N/A | N/A | 0 | 0 | 0 | 100 | N/A | N/A | N/A | N/A |
| Greinix | NR | NR | 0 | Glucksberg | N/A | N/A | N/A | N/A | 0 | 50 | 50 | 0 | N/A | N/A | N/A | N/A |
| Smith | 100 | 0 | 0 | Consensus Conf. | N/A | N/A | N/A | N/A | N/A | N/A | N/A | N/A | N/A | N/A | N/A | N/A |
| Greinix | 62 | 38 | 0 | Glucksberg | N/A | N/A | N/A | N/A | 0 | 48 | 14 | 38 | N/A | N/A | N/A | N/A |
| Salvaneschi | 100 | 0 | 0 | Glucksberg | N/A | N/A | N/A | N/A | 0 | 11 | 78 | 11 | N/A | N/A | N/A | N/A |
| Messina | 100 | 0 | 0 | Glucksberg | N/A | N/A | N/A | N/A | 6.1 | 33.3 | 39.4 | 21.2 | N/A | N/A | N/A | N/A |
| Garban | 100 | 0 | 0 | Glucksberg | N/A | N/A | N/A | N/A | N/A | * | * | 12 | N/A | N/A | N/A | N/A |
| Greinix | 63 | 37 | 0 | Glucksberg | N/A | N/A | N/A | N/A | 0 | 69 | 26 | 5 | N/A | N/A | N/A | N/A |
| Berger | 100 | 0 | 0 | Consensus Conf. | N/A | N/A | N/A | N/A | 0 | 47 | 26 | 26 | N/A | N/A | N/A | N/A |
| Kanold | 100 | 0 | 0 | NR | N/A | N/A | N/A | N/A | 0 | 25 | 50 | 25 | N/A | N/A | N/A | N/A |
| Perfetti | 82.6 | 17.4 | 0 | Consensus Conf. | N/A | N/A | N/A | N/A | 0 | 44 | 30 | 26 | N/A | N/A | N/A | N/A |
| Calore | 47 | 27 | 27 | Consensus Conf. | N/A | N/A | N/A | N/A | 0 | 47 | 27 | 27 | N/A | N/A | N/A | N/A |
| González Vicent | 100 | 0 | 0 | Glucksberg | N/A | N/A | N/A | N/A | 0 | 29 | 19 | 52 | N/A | N/A | N/A | N/A |
| Perotti | NR | NR | 0 | NR | 0 | 62 | 28 | 10 | 0 | 62 | 28 | 10 | N/A | N/A | N/A | N/A |
| Hautmann | 40 | 60 | 0 | Glucksberg | 30 | 33 | 33 | 3 | 0 | 40 | 50 | 10 | N/A | N/A | N/A | N/A |
| Jagasia | 100 | 0 | 0 | Glucksberg | N/A | N/A | N/A | N/A | * | 72 | * | 28 | N/A | N/A | N/A | N/A |
| Rubegni | 100 | 0 | 0 | Consensus Conf. | N/A | N/A | N/A | N/A | 0 | 56 | 44 | 0 | N/A | N/A | N/A | N/A |
| Ussowicz | 100 | 0 | 0 | Consensus Conf. | N/A | N/A | N/A | N/A | 0 | 0 | * | 100 | N/A | N/A | N/A | N/A |
| Das-Gupta | 61 | 39 | 0 | Consensus Conf. | N/A | N/A | N/A | N/A | 0 | 70 | * | 30 | N/A | N/A | N/A | N/A |
| Berger | NR | NR | 0 | Consensus Conf. | N/A | N/A | N/A | N/A | 0 | 47 | 35 | 18 | N/A | N/A | N/A | N/A |
| Malagola | 49 | 51 | 0 | Glucksberg | N/A | N/A | N/A | N/A | 0 | 80 | 17.8 | 2.2 | N/A | N/A | N/A | N/A |
| Niittyvuopio | 81 | 19 | 0 | Glucksberg | N/A | N/A | N/A | N/A | 6 | 14 | 65 | 15 | N/A | N/A | N/A | N/A |
| [Nygaard](https://pubmed.ncbi.nlm.nih.gov/?term=Nygaard+M&cauthor_id=29907805) | NR | NR | 0 | Consensus Conf. | N/A | N/A | N/A | N/A | 8 | 32 | 34 | 26 | N/A | N/A | N/A | N/A |
| Sakellari | 42 | 58 | 0 | Consensus Conf. | N/A | N/A | N/A | N/A | 0 | 0 | 79 | 21 | N/A | N/A | N/A | N/A |
| Worel | 100 | 0 | 0 | Consensus Conf. | N/A | N/A | N/A | N/A | 7 | 56 | 23 | 14 | N/A | N/A | N/A | N/A |
| Winther-Jørgensen | 44 | 33 | 22 | Glucksberg | N/A | N/A | N/A | N/A | 0 | 22 | 78 | 0 | N/A | N/A | N/A | N/A |
| Axt | NR | NR | 0 | Consensus Conf. | N/A | N/A | N/A | N/A | N/A | N/A | N/A | N/A | N/A | N/A | N/A | N/A |
| Oarbeascoa | NR | NR | 0 | Consensus Conf. | N/A | N/A | N/A | N/A | 0 | 29 | * | 71 | N/A | N/A | N/A | N/A |
| Modemann | 100 | 0 | 0 | Consensus Conf. | N/A | N/A | 50 | 50 | N/A | N/A | N/A | N/A | N/A | N/A | N/A | N/A |
| Batgi | 100 | 0 | 0 | Consensus Conf. | N/A | N/A | N/A | N/A | 0 | 21.3 | 28 | 46.7 | N/A | N/A | N/A | N/A |
| Kitko | 100 | 0 | 0 | IBMTR | N/A | N/A | N/A | N/A | N/A | N/A | N/A | N/A | 10.3 | 13.8 | 69 | 6.9 |
| Reschke | 100 | 0 | 0 | MAGIC | N/A | N/A | N/A | N/A | N/A | N/A | N/A | N/A | N/A | N/A | N/A | N/A |
| Canto | 39.3 | 46.4 | Contraindicated: 14.3 | MAGIC | N/A | N/A | N/A | N/A | 29.2 | 25 | 29.2 | 16.7 | N/A | N/A | N/A | N/A |
| Kaya | 100 | 0 | 0 | Glucksberg | N/A | N/A | N/A | N/A | * | 69 | * | 31 | N/A | N/A | N/A | N/A |
| [Penack](https://pubmed.ncbi.nlm.nih.gov/?term=Penack%20O%5BAuthor%5D) | 66 | 34 | 0 | Consensus Conf. | N/A | N/A | N/A | N/A | 0 | 37.7 | 35.8 | 26.4 | N/A | N/A | N/A | N/A |
| Solh | 100 | 0 | 0 | Consensus Conf. | 33 | 43 | 23 | 1 | 10 | 39 | 41 | 10 | N/A | N/A | N/A | N/A |
| Canto | 43 | 50 | 7 | MAGIC | N/A | N/A | N/A | N/A | N/A | N/A | N/A | N/A | N/A | N/A | N/A | N/A |
| Michallet | 100 | 0 | 0 | Glucksberg | N/A | N/A | N/A | N/A | 0 | 0 | 48 | 52 | N/A | N/A | N/A | N/A |

# **Supplementary Table 4 – Response to Steroids & Grade (Table S4)**

**SR –** Steroid Refractory; **SD** – Steroid Dependent; **SI** – Steroid Intolerance; **Conf.** – Conference; **N/A –** Not Applicable **(*) –** Included in the upper range

| Author(s) | Primary Disease (%) | | | | | | | | | | | | | | | | | | | | | | | | | | | | | | | | | | |
| --- | --- | --- | --- | --- | --- | --- | --- | --- | --- | --- | --- | --- | --- | --- | --- | --- | --- | --- | --- | --- | --- | --- | --- | --- | --- | --- | --- | --- | --- | --- | --- | --- | --- | --- | --- |
|  | Lymphoma | HL | NHL | NHL/HD/CLL | T-NLH | Mantle cell lymphoma | AML | ALL | AL | MCL | T-PLL | Mixed phenotype acute leukemia | Plasma cell leukemia | CML | CLL | MDS | MF | MPN | MDS  /MPS/  CML | RAEB-T | MM | Anemia | AA | Thalassemia | PNH | Cooley | Fanconi's | BM Failure | CGD | McCusick  Syndrome | Behcet | Not Malignant | KS | Solid tumor | Other |
| Besnier | N/A | N/A | N/A | N/A | N/A | N/A | N/A | N/A | N/A | N/A | N/A | N/A | N/A | N/A | N/A | N/A | N/A | N/A | N/A | N/A | N/A | N/A | N/A | N/A | N/A | N/A | N/A | N/A | N/A | N/A | N/A | N/A | N/A | N/A | N/A |
| Greinix | N/A | N/A | N/A | N/A | N/A | N/A | N/A | N/A | N/A | N/A | N/A | N/A | N/A | N/A | N/A | N/A | N/A | N/A | N/A | N/A | N/A | N/A | N/A | N/A | N/A | N/A | N/A | N/A | N/A | N/A | N/A | N/A | N/A | N/A | N/A |
| Smith | N/A | N/A | N/A | N/A | N/A | N/A | N/A | N/A | 33 | N/A | N/A | N/A | N/A | 46 | N/A | N/A | N/A | 13 | N/A | N/A | N/A | N/A | 8 | N/A | N/A | N/A | N/A | N/A | N/A | N/A | N/A | N/A | N/A | N/A | N/A |
| Greinix | N/A | N/A | N/A | N/A | N/A | N/A | 14 | 24 | N/A | N/A | N/A | N/A | N/A | 48 | N/A | N/A | N/A | N/A | N/A | N/A | N/A | N/A | N/A | N/A | N/A | N/A | N/A | N/A | N/A | N/A | N/A | N/A | N/A | N/A | 14 |
| Salvaneschi | N/A | N/A | N/A | N/A | N/A | N/A | N/A | N/A | N/A | N/A | N/A | N/A | N/A | N/A | N/A | N/A | N/A | N/A | N/A | N/A | N/A | N/A | N/A | N/A | N/A | N/A | N/A | N/A | N/A | N/A | N/A | N/A | N/A | N/A | N/A |
| Messina | N/A | N/A | 3 | N/A | N/A | N/A | 21.2 | 51.5 | N/A | N/A | N/A | N/A | N/A | 9.1 | N/A | N/A | N/A | N/A | N/A | N/A | N/A | N/A | N/A | N/A | N/A | N/A | N/A | N/A | N/A | N/A | N/A | 15.2 | N/A | N/A | N/A |
| Garban | N/A | N/A | N/A | N/A | N/A | N/A | N/A | N/A | 58 | N/A | N/A | N/A | N/A | 17 | N/A | 17 | N/A | N/A | N/A | N/A | 8 | N/A | N/A | N/A | N/A | N/A | N/A | N/A | N/A | N/A | N/A | N/A | N/A | N/A | N/A |
| Greinix | N/A | N/A | N/A | N/A | N/A | N/A | N/A | N/A | N/A | N/A | N/A | N/A | N/A | N/A | N/A | N/A | N/A | N/A | N/A | N/A | N/A | N/A | N/A | N/A | N/A | N/A | N/A | N/A | N/A | N/A | N/A | N/A | N/A | N/A | N/A |
| Berger | N/A | N/A | N/A | N/A | 7 | N/A | N/A | 40 | N/A | N/A | N/A | N/A | N/A | 27 | N/A | N/A | N/A | N/A | N/A | 7 | N/A | N/A | N/A | N/A | N/A | 13 | N/A | N/A | N/A | 7 | N/A | N/A | N/A | N/A | N/A |
| Kanold | N/A | N/A | 8.3 | N/A | N/A | N/A | 16.7 | 41.7 | N/A | N/A | N/A | N/A | N/A | N/A | N/A | N/A | N/A | N/A | N/A | N/A | N/A | N/A | 33.3 | N/A | N/A | N/A | N/A | N/A | N/A | N/A | N/A | N/A | N/A | N/A | N/A |
| Perfetti | N/A | N/A | N/A | N/A | N/A | N/A | 17.4 | 17.4 | N/A | N/A | N/A | N/A | N/A | 21.7 | N/A | 13 | 8.7 | N/A | N/A | N/A | 8.7 | N/A | 8.7 | N/A | N/A | N/A | N/A | N/A | N/A | N/A | 4..3 | N/A | N/A | N/A | N/A |
| Calore | N/A | N/A | 7 | N/A | N/A | N/A | 27 | 67 | N/A | N/A | N/A | N/A | N/A | N/A | N/A | N/A | N/A | N/A | N/A | N/A | N/A | N/A | N/A | N/A | N/A | N/A | N/A | N/A | N/A | N/A | N/A | N/A | N/A | N/A | N/A |
| González Vicent | N/A | 4 | N/A | N/A | N/A | N/A | 15 | 48 | N/A | N/A | N/A | N/A | N/A | 7 | N/A | N/A | N/A | N/A | N/A | N/A | N/A | 7 | N/A | N/A | N/A | N/A | N/A | 7 | N/A | N/A | N/A | N/A | N/A | N/A | 4 |
| Perotti | N/A | N/A | N/A | N/A | N/A | N/A | 16 | 50 | N/A | N/A | N/A | N/A | N/A | N/A | N/A | N/A | N/A | N/A | N/A | N/A | N/A | N/A | N/A | N/A | N/A | N/A | N/A | N/A | N/A | N/A | N/A | N/A | N/A | N/A | 34 |
| Hautmann | N/A | N/A | 27 | N/A | N/A | N/A | 47 | 10 | N/A | N/A | N/A | N/A | N/A | 0 | N/A | N/A | 3 | N/A | N/A | N/A | N/A | N/A | N/A | N/A | N/A | N/A | N/A | N/A | N/A | N/A | N/A | N/A | N/A | N/A | 13 |
| Jagasia | N/A | N/A | N/A | N/A | N/A | N/A | N/A | N/A | N/A | N/A | N/A | N/A | N/A | N/A | N/A | N/A | N/A | N/A | N/A | N/A | N/A | N/A | N/A | N/A | N/A | N/A | N/A | N/A | N/A | N/A | N/A | N/A | N/A | N/A | N/A |
| Rubegni | N/A | N/A | 22 | N/A | N/A | N/A | 11 | N/A | N/A | N/A | N/A | N/A | N/A | 11 | 33 | N/A | N/A | N/A | N/A | N/A | 22 | N/A | N/A | N/A | N/A | N/A | N/A | N/A | N/A | N/A | N/A | N/A | N/A | N/A | N/A |
| Ussowicz | N/A | N/A | 12.5 | N/A | N/A | N/A | 12.5 | 37.5 | N/A | N/A | N/A | N/A | N/A | 25 | N/A | N/A | N/A | N/A | N/A | N/A | N/A | N/A | 12.5 | N/A | N/A | N/A | N/A | N/A | N/A | N/A | N/A | N/A | N/A | N/A | N/A |
| Das-Gupta | N/A | N/A | N/A | N/A | N/A | N/A | N/A | N/A | N/A | N/A | N/A | N/A | N/A | N/A | N/A | N/A | N/A | N/A | N/A | N/A | N/A | N/A | N/A | N/A | N/A | N/A | N/A | N/A | N/A | N/A | N/A | N/A | N/A | N/A | N/A |
| Berger | 9 | N/A | N/A | N/A | N/A | N/A | 6 | 38 | N/A | N/A | N/A | N/A | N/A | 18 | N/A | 6 | N/A | N/A | N/A | N/A | 0 | N/A | N/A | N/A | N/A | N/A | N/A | N/A | N/A | N/A | N/A | 18 | N/A | 6 | N/A |
| Malagola | N/A | 8.89 | 8.89 | N/A | N/A | N/A | 28.89 | 22.22 | N/A | N/A | N/A | N/A | N/A | N/A | N/A | 13.33 | N/A | N/A | N/A | N/A | 13.33 | N/A | N/A | N/A | N/A | N/A | N/A | N/A | N/A | N/A | N/A | N/A | N/A | N/A | 6 |
| Niittyvuopio | N/A | N/A | 13.5 | N/A | N/A | N/A | 21.2 | 13.5 | N/A | N/A | 1.9 | N/A | N/A | 11.5 | 3.9 | 13.5 | 9.6 | N/A | N/A | N/A | 11.5 | N/A | N/A | N/A | N/A | N/A | N/A | N/A | N/A | N/A | N/A | N/A | N/A | N/A | N/A |
| Nygaard | N/A | N/A | N/A | N/A | N/A | N/A | N/A | N/A | N/A | N/A | N/A | N/A | N/A | N/A | N/A | N/A | N/A | N/A | N/A | N/A | N/A | N/A | N/A | N/A | N/A | N/A | N/A | N/A | N/A | N/A | N/A | N/A | N/A | N/A | N/A |
| Sakellari | N/A | 5 | 16 | N/A | N/A | N/A | 26 | 26 | N/A | N/A | N/A | N/A | N/A | N/A | N/A | 5 | N/A | 5 | N/A | N/A | 16 | N/A | N/A | N/A | N/A | N/A | N/A | N/A | N/A | N/A | N/A | N/A | N/A | N/A | N/A |
| Worel | N/A | N/A | N/A | N/A | N/A | N/A | 47 | 18 | N/A | N/A | N/A | N/A | N/A | 15 | N/A | 7 | N/A | N/A | N/A | N/A | N/A | N/A | N/A | N/A | N/A | N/A | N/A | N/A | N/A | N/A | N/A | N/A | N/A | N/A | 13 |
| Winther-Jørgensen | N/A | N/A | 11 | N/A | N/A | N/A | 22 | 22 | N/A | N/A | N/A | N/A | N/A | 11 | N/A | N/A | N/A | N/A | N/A | N/A | N/A | N/A | 11 | N/A | N/A | N/A | 11 | N/A | N/A | N/A | N/A | N/A | 11 | N/A | N/A |
| Axt | N/A | N/A | N/A | N/A | N/A | N/A | N/A | N/A | N/A | N/A | N/A | N/A | N/A | N/A | N/A | N/A | N/A | N/A | N/A | N/A | N/A | N/A | N/A | N/A | N/A | N/A | N/A | N/A | N/A | N/A | N/A | N/A | N/A | N/A | 0.8 |
| Oarbeascoa | N/A | N/A | N/A | N/A | N/A | N/A | 37 | 12 | N/A | N/A | N/A | N/A | N/A | N/A | N/A | 9 | N/A | N/A | N/A | N/A | N/A | N/A | N/A | N/A | N/A | N/A | N/A | N/A | N/A | N/A | N/A | N/A | N/A | N/A | 42 |
| Modemann | N/A | N/A | N/A | N/A | N/A | N/A | 11 | 17 | N/A | N/A | N/A | N/A | N/A | 6 | N/A | 33 | 28 | N/A | N/A | N/A | 6 | N/A | N/A | N/A | N/A | N/A | N/A | N/A | N/A | N/A | N/A | N/A | N/A | N/A | N/A |
| Batgi | N/A | 2.6 | 2.6 | N/A | N/A | N/A | 42.66 | 30.6 | N/A | N/A | N/A | N/A | N/A | N/A | N/A | 4 | N/A | N/A | N/A | N/A | 2.6 | N/A | 2.6 | N/A | N/A | N/A | N/A | N/A | N/A | N/A | N/A | N/A | N/A | N/A | 12 |
| Kitko | N/A | N/A | N/A | N/A | N/A | N/A | 24.1 | 34.5 | N/A | N/A | N/A | N/A | N/A | N/A | N/A | N/A | N/A | N/A | N/A | N/A | N/A | N/A | N/A | N/A | N/A | N/A | N/A | N/A | N/A | N/A | N/A | 27.6 | N/A | N/A | 13.8 |
| Reschke | N/A | N/A | 22.22 | N/A | N/A | N/A | N/A | N/A | N/A | 11.11 | N/A | N/A | N/A | N/A | 11.11 | 33.33 | N/A | N/A | N/A | N/A | 22.22 | N/A | N/A | N/A | N/A | N/A | N/A | N/A | N/A | N/A | N/A | N/A | N/A | N/A | N/A |
| Canto | N/A | N/A | N/A | N/A | N/A | N/A | 27.5 | 55.2 | N/A | N/A | N/A | N/A | N/A | 6.9 | N/A | N/A | N/A | N/A | N/A | N/A | N/A | N/A | N/A | N/A | N/A | N/A | 3.4 | N/A | 3.4 | N/A | N/A | N/A | N/A | N/A | 3.4 |
| Kaya | N/A | 3 | 3 | N/A | N/A | N/A | 31 | 34 | N/A | N/A | N/A | N/A | N/A | N/A | N/A | N/A | N/A | N/A | N/A | N/A | 3 | N/A | 12 | 9 | 6 | N/A | N/A | N/A | N/A | N/A | N/A | N/A | N/A | N/A | N/A |
| Penack | 9.4 | N/A | N/A | N/A | N/A | N/A | N/A | N/A | 56.6 | N/A | N/A | N/A | N/A | N/A | 7.5 | 26.4 | N/A | N/A | N/A | N/A | N/A | N/A | N/A | N/A | N/A | N/A | N/A | N/A | N/A | N/A | N/A | N/A | N/A | N/A | N/A |
| Solh | N/A | N/A | N/A | 19 | N/A | N/A | 29 | 19 | N/A | N/A | N/A | N/A | N/A | N/A | N/A | N/A | N/A | N/A | 29 | N/A | N/A | N/A | N/A | N/A | N/A | N/A | N/A | N/A | N/A | N/A | N/A | N/A | N/A | N/A | 4 |
| Canto | N/A | 14.2 | N/A | N/A | N/A | 3.6 | 35.7 | 14.3 | N/A | N/A | N/A | 7.1 | 3.6 | 3.6 | N/A | 7.1 | 7.1 | N/A | N/A | N/A | 3.6 | N/A | N/A | N/A | N/A | N/A | N/A | N/A | N/A | N/A | N/A | N/A | N/A | N/A | N/A |
| Michallet | N/A | N/A | 3.5 | N/A | N/A | N/A | 30 | 15 | N/A | N/A | N/A | N/A | N/A | 4.5 | 8.5 | 17 | N/A | 9.5 | N/A | N/A | 12 | N/A | N/A | N/A | N/A | N/A | N/A | N/A | N/A | N/A | N/A | N/A | N/A | N/A | N/A |

# **Supplementary Table 5 – Primary Disease (Table S5)**

**HL –** Hodgkin’s Lymphoma**; NHL –** Non-Hodgkin’s Lymphoma**; AML –** Acute Myeloid Leukemia**; ALL –** Acute Lymphoblastic Leukemia**; AL –** Acute Leukemia**; MCL –** Mantle Cell Lymphoma**; T-PLL –** T-cell Prolymphocytic Leukemia**; CML –** Chronic Myeloid Leukemia**; CLL –** Chronic Lymphocytic Leukemia**; CL –** Chronic Leukemia**; MDS –** Myelodysplastic Syndrome**; MF –** Myelofibrosis**; MPN –** Myeloproliferative Neoplasm**; RAEB-T –** Refractory Anemia with Excess Blasts in Transformation**; MM** – Multiple Myeloma**; AA –** Aplastic Anemia**; SAA** – Severe Aplastic Anemia**; PNH –** Paroxysmal Nocturnal Hemoglobinuria**; BM Failure –** Bone Marrow Failure**; CGD –** Chronic Granulomatous Disease

| Author(s) | Conditioning Regimen (n) | | | | | | | | |
| --- | --- | --- | --- | --- | --- | --- | --- | --- | --- |
|  | Group A | Group B | Group C | Group D | Group E | Group F | Group G | Group H | Group I |
| Besnier | NR | NR |  |  |  |  |  |  |  |
| Greinix | MAC: 100 | NR | NR |  |  |  |  |  |  |
| Smith | fTBI and etoposide: 46 | fTBI and CTX: 21 | fTBI, CTX, and etoposide: 12.5 | CTX and BU: 12.5 | TLI and CTX: 4 | CTX: 4 |  |  |  |
| Greinix | CTX, fTBI: 90 | Bu, CTX: 10 | NR |  |  |  |  |  |  |
| Salvaneschi | NR | NR | NR |  |  |  |  |  |  |
| Messina | NR | NR | NR |  |  |  |  |  |  |
| Garban | MAC :83 | RIC: 17 | NMA: 0 |  |  |  |  |  |  |
| Greinix | NR | NR | NR |  |  |  |  |  |  |
| Berger | TBI 1200, CTX 120: 20 | TBI 1200, CTX 120, Etoposide: 7 | TBI 1200, CTX 100, TT 10: 13 | TBI 1200, CTX 120, TT 10: 20 | BU 16, CTX 120, Mel 140: 7 | BU 16, CTX 200: 7 | Mel 160, Flu 200, TT 10: 7 | BU 14, TT 6, Flu 120: 13 | TT 5, Flu 120, Mel 140: 7 |
| Kanold | NR | NR | NR |  |  |  |  |  |  |
| Perfetti | MAC: 43.5 | RIC: 56.5 | NMA: 0 |  |  |  |  |  |  |
| Calore | TBI: 80 | BU+CTX: 7 | Flu+Mel: 13 |  |  |  |  |  |  |
| González Vicent | MAC: 56 | RIC: 44 | NMA: 0 |  |  |  |  |  |  |
| Perotti | NR | NR |  |  |  |  |  |  |  |
| Hautmann | NR | NR | NR |  |  |  |  |  |  |
| Jagasia | MAC: 68 | RIC/NMA: 32 | NR |  |  |  |  |  |  |
| Rubegni | Mel, Flu: 22 | Mel, Flu, CARMU: 22 | Flu, CTX: 44 |  |  |  |  |  |  |
| Ussowicz | NR | NR | NR |  |  |  |  |  |  |
| Das-Gupta | MAC: 77 | RIC/NMA: 23 | NR |  |  |  |  |  |  |
| Berger | MAC: 76 | NR | NMA: 23 |  |  |  |  |  |  |
| Malagola | MAC: 44 | RIC: 56 | NMA: 0 |  |  |  |  |  |  |
| Niittyvuopio | MAC: 75 | RIC: 25 | NMA: 0 |  |  |  |  |  |  |
| [Nygaard](https://pubmed.ncbi.nlm.nih.gov/?term=Nygaard+M&cauthor_id=29907805) | MAC: 34 | RIC: 0 | NMA: 66 | 1200Gy: 9 | No 1200Gy: 91 |  |  |  |  |
| Sakellari | MAC: 58 | RIC: 42 |  |  |  |  |  |  |  |
| Worel | MAC: 77 | RIC: 23 |  |  |  |  |  |  |  |
| Winther-Jørgensen | Bu, Etoposide, CTX: 11 | Clo, TT, Mel: 11 | Bu, CTX, Mel: 11 | TBI, Etoposide: 11 | Treo, TT, Flu: 11 | Flu, Thio, CTX, Alem: 11 | CTX, Flu: 11 | Flu, Thio, Mel: 11 | Bu, CTX: 11 |
| Axt | NR | NR |  |  |  |  |  |  |  |
| Oarbeascoa | MAC: 55 | RIC: 45 | NMA: 0 |  |  |  |  |  |  |
| Modemann | NR | NR | NR |  |  |  |  |  |  |
| Batgi | MAC: 93.3 | RIC: 6.7 | NMA: 0 |  |  |  |  |  |  |
| Kitko | NR | NR | NR |  |  |  |  |  |  |
| Reschke | 2Gy, Flu: 55.56 | CTX, BU, Flu, ATG: 11.11 | 12Gy, CTX, ATG: 11.11 | Bu, Flu, ATG, Phen: 11.11 | Bu, Flu: 11.11 |  |  |  |  |
| Canto | NR | NR |  |  |  |  |  |  |  |
| Kaya | MAC: 100 | RIC: 0 | NMA: 0 |  |  |  |  |  |  |
| [Penack](https://pubmed.ncbi.nlm.nih.gov/?term=Penack%20O%5BAuthor%5D) | MAC: 58.8 | RIC: 41.2 | NMA: 0 |  |  |  |  |  |  |
| Solh | MAC: 44 | RIC: 24 | NMA: 32 |  |  |  |  |  |  |
| Canto | MAC: 39.3 | RIC: 60.7 | NMA: 0 |  |  |  |  |  |  |
| Michallet | MAC: 38 | NR | NMA:62 |  |  |  |  |  |  |

# **Supplementary Table 6 – Conditioning Regimen (Table S6)**

**MAC** – Myeloablative Conditioning; **RIC** – Reduced Intensity Conditioning; **NMA** – Non-myeloablative Conditioning; **TBI** – Total Body Irradiation; **fTBI** – Fractioned total body irradiation; **CTX** – Cyclophosphamide; **TLI** – Total Lymphoid Irradiation; **BU** – Busulfan; **TT** – Thiotepa; **Treo** - Treosulfan; **Mel** – Melphalan; **Flu** – Fludarabine; **CARMU** – Carmustine; **ATG** – Anti-Thymocyte Globulin; **Phen** – Phenytoin; **Gy** – Gray; **NR** – Not reported; **N/A** – Not available.

| Author(s) | Treatment Combination | Percentage | Treatment Combination | Percentage | Treatment Combination | Percentage | Treatment Combination | Percentage | Treatment Combination | Percentage | Treatment Combination | Percentage |
| --- | --- | --- | --- | --- | --- | --- | --- | --- | --- | --- | --- | --- |
| Besnier | None |  |  |  |  |  |  |  |  |  |  |  |
| Greinix | CsA, Steroid | 100 |  |  |  |  |  |  |  |  |  |  |
| Smith | ATG | 33 |  |  |  |  |  |  |  |  |  |  |
| Greinix | CsA, Steroid | 100 |  |  |  |  |  |  |  |  |  |  |
| Salvaneschi | CsA, Steroid | N/A | Steroid, Tac/FK | N/A | Steroid | N/A |  |  |  |  |  |  |
| Messina ** | Steroid | N/A | NM |  |  |  |  |  |  |  |  |  |
| Garban | CsA, Steroid | N/A | CsA, MMF, Steroid | N/A |  |  |  |  |  |  |  |  |
| Greinix | CsA, Steroid | 100 |  |  |  |  |  |  |  |  |  |  |
| Berger ** | NR |  |  |  |  |  |  |  |  |  |  |  |
| Kanold | Steroid | 100 |  | N/A |  | N/A |  |  |  |  |  |  |
| Perfetti * | CsA, Steroid | 73.9 | CsA, Steroid, ATG | 8.7 | CsA, Steroid, MTX | 4.3 | Steroid, Tac/FK | 4.3 | Steroid | 4.3 | MMF | 4.3 |
| Calore ** | NR |  |  |  |  |  |  |  |  |  |  |  |
| González Vicent | None |  |  |  |  |  |  |  |  |  |  |  |
| Perotti | NR | N/A |  |  |  |  |  |  |  |  |  |  |
| Hautmann * | CNI, Steroid, MMF | 37 | Steroid, CNI | 7 | Other | 47 |  |  |  |  |  |  |
| Jagasia | Steroid | 100 |  |  |  |  |  |  |  |  |  |  |
| Rubegni * | Steroid, CsA | 67 | MMF, Steroid, CsA | 11 | Steroid | 11 | MMF, Eta, CsA | 11 |  |  |  |  |
| Ussowicz * | Steroid |  | ETA |  | basiliximab |  |  |  |  |  |  |  |
| Das-Gupta | Steroid | 100 |  |  |  |  |  |  |  |  |  |  |
| Berger ** | Steroid | 100 | NM |  |  |  |  |  |  |  |  |  |
| Malagola | Steroid | 100 |  |  |  |  |  |  |  |  |  |  |
| Niittyvuopio * | Pentostatin, Steroid | 19.2 | ATG, Alemtuzumab, Infliksimab, Steroid | 15.4 | Steroid | 65.4 |  |  |  |  |  |  |
| [Nygaard](https://pubmed.ncbi.nlm.nih.gov/?term=Nygaard+M&cauthor_id=29907805) * | Infliximab, Steroid | 71 | Steroid | 29 |  |  |  |  |  |  |  |  |
| Sakellari * | ATG | 42 | NM | 58 |  |  |  |  |  |  |  |  |
| Worel | Steroid | 100 |  |  |  |  |  |  |  |  |  |  |
| Winther-Jørgensen * | Steroid | 11 | Steroid ,Tac/FK | 22 | Steroid ,CsA | 22 | Infliximab, Steroid, CsA | 11 | Infliximab, Steroid, MMF, Tac/FK | 22 | Steroid, Tac/FK, CsA | 11 |
| Axt | NR |  |  |  |  |  |  |  |  |  |  |  |
| Oarbeascoa | Steroid | 100 |  |  |  |  |  |  |  |  |  |  |
| Modemann * | Ruxolitinib, Steroid, CNI | 6 | Ruxolitinib, Steroid, CNI, MMF | 94 |  |  |  |  |  |  |  |  |
| Batgi | Steroid | 100 |  |  |  |  |  |  |  |  |  |  |
| Kitko | Steroid | 100 |  | N/A |  | N/A |  |  |  |  |  |  |
| Reschke | Steroid | 100 |  |  |  |  |  |  |  |  |  |  |
| Canto ** | NR |  |  |  |  |  |  |  |  |  |  |  |
| Kaya | Steroid | 100 |  | N/A |  | N/A |  |  |  |  |  |  |
| [Penack](https://pubmed.ncbi.nlm.nih.gov/?term=Penack%20O%5BAuthor%5D) * | Steroid | 43.4 | Steroid, CNI | 41.5 | MMF, Steroid | 9.4 | Steroid, Sir | 9.4 | Steroid, Other | 9.4 |  |  |
| Solh * | NR | 56 | Ruxolitinib | 16 | Infliximab/basiliximab ± rituximab | 28 |  |  |  |  |  |  |
| Canto | Steroid | 100 | Steroid | 100 |  |  |  |  |  |  |  |  |
| Michallet | NR | N/A |  |  |  |  |  |  |  |  |  |  |

# **Supplementary Table 7 – Treatment Combination (Table S7)**

**CsA** – Cyclosporine A; **ATG** – Anti-Thymocyte Globulin; **Tac/FK** – Tacrolimus; **MMF** – Mycophenolate, **CNI** – Calcineurin Inhibitor; **Eta** – Etanercept, **NR** – Not reported; **N/A** – Not available.

**(*) –** Considered to be in combination with an active drug

**(**)** – Unknown combination

| Author(s) | Donor Type (n) | | | | | | | | Source (%) | | |
| --- | --- | --- | --- | --- | --- | --- | --- | --- | --- | --- | --- |
|  | Group A | Group B | Group C | Group D | Group E | Group F | Group G | Group H | Cord Blood | Peripheral | Bone Marrow |
| Besnier | MRD: 23 | MUD: 32 |  |  |  |  |  |  | NR | NR | NR |
| Greinix | NR | NR | NR | NR | NR | NR |  |  | 0 | 0 | 100 |
| Smith | HLA·ldentical sibling: 33 | 5/6 HLA sibling: 17 | URD: 50 |  |  |  |  |  | NR | NR | NR |
| Greinix | Related: 28 | URD: 72 | Serologic and LBT match: 81 | Serologic class I mismatch: 19 |  |  |  |  | 0 | 10 | 90 |
| Salvaneschi | Sibling: 33 | MUD: 56 | Partially matched family donor: 11 | NR |  |  |  |  | NR | NR | NR |
| Messina | MRD: 27.3 | MUD: 72.7 |  |  |  |  |  |  | 6.1 | 6.1 | 87.8 |
| Garban | Matched sibling: 67 | Unrelated: 33 | NR | NR |  |  |  |  | 0 | 75 | 25 |
| Greinix | NR | NR | NR | NR | NR | NR |  |  | NR | NR | NR |
| Berger | MRD: 33 | MUD: 67 |  |  |  |  |  |  | 13 | 13 | 73 |
| Kanold | NR | NR | NR | NR | NR | NR |  |  | 0 | 0 | 100 |
| Perfetti |  | MUD: 52.2 | MMUD: 34.8 | MMRD: 13 |  |  |  |  | 0 | 13 | 87 |
| Calore | Matched: 60 | Mismatched (5/6): 40 |  |  |  |  |  |  | NR | NR | NR |
| González Vicent | PBSC: 56 | BM: 26 | CB: 18 |  |  |  |  |  | 18 | 56 | 26 |
| Perotti | Related: 24 | Unrelated: 64 | Haplo:12 |  |  |  |  |  | NR | NR | NR |
| Hautmann | Related: 43 | Unrelated: 57 | NR | NR |  |  |  |  | NR | NR | NR |
| Jagasia | Related: 21 | Unrelated: 79 | 10/10: 83 | Other: 17 |  |  |  |  | 5 | 84 | 11 |
| Rubegni | HLA-identical siblings: 89 | Unrelated Donor: 11 | Sex Matched: 75 | Non-sex Matched: 25 |  |  |  |  | 0 | 44 | 56 |
| Ussowicz | MSD: 25 | MUD: 75 |  |  |  |  |  |  | NR | NR | NR |
| Das-Gupta | Related: 77 | Unrelated: 23 | 10/10: 69 | Other: 31 |  |  |  |  | 6 | 60 | 34 |
| Berger | MRD: 23 | MUD: 76 | Female donor/male recipient: 20 | Other: 79 | Major incompatibility: 38 | Minor incompatibility: 20 | Identical: 41 |  | 9 | 26 | 65 |
| Malagola | Sibling: 70 | MUD: 22 | 6/6: 90 | 5/6: 4 | 4/6: 6 | CB: 8 |  |  | 0 | 84 | 16 |
| Niittyvuopio | Related: 40.2 | Unrelated: 57.7 | dCB: 2 |  |  |  |  |  | 2 | 79 | 19 |
| [Nygaard](https://pubmed.ncbi.nlm.nih.gov/?term=Nygaard+M&cauthor_id=29907805) | Related: 18 | Unrelated: 82 |  |  |  |  |  |  | 0 | 84 | 16 |
| Sakellari | Sibling: 21 | Haploidentical: 5 | 8/8 HLA-matched unrelated: 32 | 7/8 HLA-matched unrelated: 42 |  |  |  |  | 0 | 21 | 79 |
| Worel | Related: 16 | Unrelated: 84 | HLA identical: 71 | HLA mismatch: 1 locus: 25 | Missing: 4 |  |  |  | 3 | 83 | 14 |
| Winther-Jørgensen | MUD: 44 | MFD: 22 | Haplo, T‐cell depleted: 33 |  |  |  |  |  | NR | NR | NR |
| Axt | NR | NR | NR | NR | NR | NR |  |  | NR | NR | NR |
| Oarbeascoa | MRD: 34 | MUD: 20 | MMUD: 5 | MMRD: 3 | CB: 1 | Haploidentical related: 37 |  |  | 2 | 86 | 12 |
| Modemann | MRD: 17 | MUD: 50 | MMUD 9/10: 33 |  |  |  |  |  | 0 | 100 | 0 |
| Batgi | MRD: 68 | 1 MMRD: 3 | 2 MMRD: 1 | MUD: 23 | 1 Mismatched unrelated donor: 5 |  |  |  | 0 | 100 | 0 |
| Kitko | MRD: 13.8 | MUD: 62.1 | MMUD: 6.9 | MMRD: 17.2 |  |  |  |  | 3.4 | 24.1 | 72.4 |
| Reschke | Identical HLA: 78 | Different HLA: 22 |  |  |  |  |  |  | NR | NR | NR |
| Canto | MUD: 48.3 | MSD: 6.9 | Haplo: 37.9 | CB: 6.9 |  |  |  |  | NR | NR | NR |
| Kaya | Related: 66 | Non-related: 34 | 8/8: 97 | 8/7: 3 |  |  |  |  | 0 | 100 | 0 |
| [Penack](https://pubmed.ncbi.nlm.nih.gov/?term=Penack%20O%5BAuthor%5D) | MRD: 23.7 | MUD 10/10: 30.2 | MMUD 9/10: 17 | MMUD 8/10 or less: 1.9 | UD (unknown mismatch): 3.8 |  |  |  | 0 | 81.1 | 18.9 |
| Solh | MRD: 28 | MUD: 40 | Haplo: 32 |  |  |  |  |  | 4 | 77 | 19 |
| Canto | HLA-identical: 46.4 | Haploidentical: 28.6 | Cord Blood: 14.2 | HLA-mismatched: 10.7 |  |  |  |  | 14.2 | 75 | 10.7 |
| Michallet | Identical Siblings; 51 | MRD: 1 | MUD: 24 | MMUD: 24 |  |  |  |  | 8 | 65 | 27 |

# **Supplementary Table 8 – Donor Type & Source (Table S8)**

**MRD** – Matched Related Donor; **MSD** – Matched Sibling Donor; **MUD –** Matched Unrelated Donor; **Haplo** – Haploidentical; **CB** – Cord Blood; **DCB** – Double-Unit Cord Blood; **MMRD** – Mismatched Related Donor; **MMUD –** Mismatched Unrelated Donor; **HLA** – Human Leukocyte Antigen; **NR** – Not Reported

| **Study ID: Author name (year)** | **Newcastle-Ottowa scale for cohort** | | | | | | | | |
| --- | --- | --- | --- | --- | --- | --- | --- | --- | --- |
|  | **Selection** | | | | **Comparability** | **Outcome** | | | **Score (Out of 9)** |
|  | Representativeness of the exposed cohort (*) | Selection of the non-exposed cohort (*) | Ascertainment of exposure (*) | Demonstration that outcome of interest was not present at start of study (*) | Comparability of cohorts on the basis of the design or analysis (* *) | Assessment of outcome (*) | Was follow-up long enough for outcomes to occur (*) | Adequacy of follow up of cohorts (*) |  |
| **Jagasia (2013)** | * | X | * | * | * | * | * | * | 7 |
| **Solh (2023)** | * | * | * | * | * | * | * | * | 8 |
| **Penack (2023)** | * | * | * | * | * * | * | * | * | 9 |

# **Supplementary Table 9 – Quality assessment for controlled retrospective cohort studies (Table S9)**

| **Study ID: Author name (year)** | **Modified Newcastle-Ottowa scale for cohort** | | | | | | |
| --- | --- | --- | --- | --- | --- | --- | --- |
|  | **Selection** | | | **Outcome** | | | **Score (Out of 6 )** |
|  | Representativeness of the exposed cohort (*) | Ascertainment of exposure (*) | Demonstration that outcome of interest was not present at start of study (*) | Assessment of outcome (*) | Was follow-up long enough for outcomes to occur (*) | Adequacy of follow up of cohorts (*) |  |
| **Besnier (1997)** | * | * | * | * | * | * | 6 |
| **Greinix (1998)** | * | * | * | * | - | * | 5 |
| **Messina (2003)** | - | * | * | * | * | * | 5 |
| **Garban (2005)** | * | * | * | ? | ? | * | 4 |
| **Berger Massimo (2007)** | * | * | * | * | * | - | 5 |
| **Calore (2008)** | * | * | - | * | * | - | 4 |
| **Perfetti (2008)** | * | * | * | * | * | * | 6 |
| **Perotti (2010)** | * | * | * | * | * | * | 6 |
| **González Vicent (2010)** | * | * | * | * | - | * | 5 |
| **Hautmann (2013)** | * | * | * | * | * | * | 6 |
| **Rubegni (2013)** | * | * | * | * | * | * | 6 |
| **Ussowicz (2013)** | * | * | * | * | * | * | 6 |
| **Das Gupta (2014)** | * | * | * | * | * | * | 6 |
| **Berger (2015)** | * | * | * | * | * | * | 6 |
| **Malagola (2016)** | - | * | * | * | * | * | 5 |
| **Sakellari (2018)** | * | * | * | * | * | * | 6 |
| **Nygaard (2018)** | * | * | * | * | * | * | 6 |
| **Niittyvuopio (2018)** | * | * | * | * | * | * | 6 |
| **Worel (2018)** | * | * | * | * | * | * | 6 |
| **Axt (2019)** | * | * | * | * | - | * | 5 |
| **Winther-Jørgensen (2019)** | * | * | * | * | * | * | 6 |
| **Modemann (2020)** | * | * | * | * | * | * | 6 |
| **Oarbeascoa (2020)** | * | * | * | * | * | * | 6 |
| **Batgi (2021)** | * | * | * | * | - | * | 5 |
| **Reschke (2022)** | * | * | * | * | - | * | 5 |
| [**Asensi Cantó (2022)**](https://doi.org/10.1002/jca.22012) | * | * | * | * | - | * | 5 |
| **Kaya (2023)** | - | * | * | * | * | * | 5 |
| **Asensi Cantó (2023)** | * | * | * | * | * | * | 6 |
| **Michallet (2024)** | * | * | * | * | * | * | 6 |

# **Supplementary Table 10 – Quality assessment for single-arm retrospective cohort studies (Table S10)**

| **Study ID: Author name (year)** | **Methodological index for non-randomized studies (MINORS)** | | | | | | | | **Score (Out of 16 )** |
| --- | --- | --- | --- | --- | --- | --- | --- | --- | --- |
|  | A clearly stated aim | Inclusion of consecutive patients | Prospective collection of data | Endpoints appropriate to the aim of the study | Unbiased assessment of the study endpoint | Follow-up period appropriate to the aim of the study | Loss to follow up less than 5% | Prospective calculation of the study size |  |
| **Smith (1998)** | **2** | **2** | **2** | **2** | **2** | **2** | **2** | **0** | **14** |
| **Greinix (2000)** | **2** | **1** | **0** | **2** | **0** | **2** | **2** | **0** | **9** |
| **Salvaneschi (2001)** | **2** | **2** | **2** | **2** | **1** | **2** | **2** | **0** | **13** |
| **Kanold (2007)** | **2** | **2** | **2** | **2** | **2** | **2** | **2** | **0** | **14** |
| **Greinix (2006)** | **2** | **2** | **0** | **2** | **0** | **2** | **2** | **0** | **10** |
| **Kitko (2022)** | **2** | **2** | **2** | **2** | **2** | **0** | **1** | **0** | **11** |

# **Supplementary Table 11 – Quality assessment for single-arm interventional studies (Table S11)**
